# Dietary Caprylic Acid (C8:0) Does Not Increase Plasma Acylated Ghrelin but Decreases Plasma Unacylated Ghrelin in the Rat

**DOI:** 10.1371/journal.pone.0133600

**Published:** 2015-07-21

**Authors:** Fanny Lemarié, Erwan Beauchamp, Stéphanie Dayot, Cécile Duby, Philippe Legrand, Vincent Rioux

**Affiliations:** Laboratory of Biochemistry and Human Nutrition, Agrocampus Ouest-INRA (USC 1378), Rennes, France; National Institute of Agronomic Research, FRANCE

## Abstract

Focusing on the caprylic acid (C8:0), this study aimed at investigating the discrepancy between the formerly described beneficial effects of dietary medium chain fatty acids on body weight loss and the C8:0 newly reported effect on food intake *via* ghrelin octanoylation. During 6 weeks, Sprague-Dawley male rats were fed with three dietary C8:0 levels (0, 8 and 21% of fatty acids) in three experimental conditions (moderate fat, caloric restriction and high fat). A specific dose-response enrichment of the stomach tissue C8:0 was observed as a function of dietary C8:0, supporting the hypothesis of an early preduodenal hydrolysis of medium chain triglycerides and a direct absorption at the gastric level. However, the octanoylated ghrelin concentration in the plasma was unchanged in spite of the increased C8:0 availability. A reproducible decrease in the plasma concentration of unacylated ghrelin was observed, which was consistent with a decrease in the stomach preproghrelin mRNA and stomach ghrelin expression. The concomitant decrease of the plasma unacylated ghrelin and the stability of its acylated form resulted in a significant increase in the acylated/total ghrelin ratio which had no effect on body weight gain or total dietary consumption. This enhanced ratio measured in rats consuming C8:0 was however suspected to increase (i) growth hormone (GH) secretion as an increase in the GH-dependent mRNA expression of the insulin like growth Factor 1 (IGF-1) was measured (ii) adipocyte diameters in subcutaneous adipose tissue without an increase in the fat pad mass. Altogether, these results show that daily feeding with diets containing C8:0 increased the C8:0 level in the stomach more than all the other tissues, affecting the acylated/total ghrelin plasma ratio by decreasing the concentration of circulating unacylated ghrelin. However, these modifications were not associated with increased body weight or food consumption.

## Introduction

Caprylic acid (octanoic acid, C8:0) belongs to the class of medium-chain saturated fatty acids (MCFAs) which also includes caproic acid (C6:0) and capric acid (C10:0). MCFAs are characteristic nutrients present in dairy products [1] and in specific oils like palm kernel and coconut oils [2]. They display physical and metabolic properties that are distinct from those of long-chain saturated fatty acids (LCFAs ≥12 carbons), leading therefore to distinct physiological effects [3]. First, part of MCFAs coming from dietary medium chain triglycerides (MCTs) can be released early during digestion through the action of preduodenal lipase [4], leading to their potential and yet not clearly quantified direct absorption by the stomach mucosa [5,6]. Second, MCFAs which have escaped the absorption at the gastric level are absorbed by small intestinal cells, like LCFAs, after the subsequent action of duodenal pancreatic lipase on both dietary remaining MCTs and long chain triglycerides (LCTs). However, unlike LCFAs which are re-esterified with 2-monoglycerides into triglycerides and incorporated into chylomicrons which enter the lymphatic system, MCFAs are directly transferred to the portal circulation and transported as free fatty acids (FFAs) with albumin to the liver [7]. Third, once in the liver, MCFAs are rapidly subjected to mitochondrial beta-oxidation [8], since they easily enter the mitochondria independently of the carnitine transport system, as opposed to LCFAs [9].

These metabolic properties of MCFAs (rapid gastro-intestinal hydrolysis and absorption, specific transport through the portal vein and rapid beta-oxidation in the liver) lead to a high catabolism and low tissue storage especially in adipose tissue [10]. Dietary MCFAs have therefore been associated with beneficial physiological effects compared with LCFAs. Indeed, a diminished deposition of fat was reported in rats overfed with MCT diets compared with LCT diets [11–13]. In overweight humans, intakes of equal-caloric diets rich in MCFAs were shown to decrease adiposity and increase energy expenditure compared to similar diets rich in LCFAs [14,15]. Regarding other physiological parameters, epidemiological studies have also shown that intakes of short to medium-chain saturated fatty acids were not significantly associated with the risk of coronary heart disease (CHD) [16].

However, more recently, caprylic acid was also shown to specifically acylate ghrelin [17], the only known peptide hormone with an orexigenic effect. Ghrelin is a 28 amino acid peptide expressed mainly in the different sections of the digestive tract and especially the stomach [18]. Acylated ghrelin binds to the growth hormone secretagogue receptor 1a (GHSR-1a) located in the pituitary gland and hypothalamus [19,20] and regulates many relevant biological processes including the secretion of the growth hormone (GH), the stimulation of appetite and food intake, the modulation of gastric acid secretion and motility and the regulation of glucose homeostasis and adiposity [21]. Ghrelin seems to have an essential function in blood glucose regulation in case of calorie restriction [22], even if the mechanisms mediating this effect remain poorly understood. During its maturation in the gastric mucosa and before secretion in the blood, part of the proghrelin is subjected to a unique modification consisting in the addition of an activated caprylic acyl-coA to the 3^rd^ serine residue. The ghrelin O-acyltransferase (GOAT or MBOAT4), the gastric enzyme involved in ghrelin octanoylation, belongs to the family of membrane bound O-acyltransferases (MBOAT), a group of proteins involved in acetyltransferase and acyltransferase activity [23,24]. Plasma ghrelin exists therefore in both unacylated and acylated forms, but only the latter can bind its GHSR-1a receptor. Because of the above-mentioned potential specific gastric absorption of MCFAs following the consumption of MCTs, dietary caprylic acid is now suspected to directly provide GOAT enzyme with octanoyl-CoA co-substrate necessary for the acyl modification of ghrelin. Indeed, ingestion by mice of either MCFAs or MCTs increased the stomach concentration of acylated ghrelin [25], without changing the total ghrelin amount. Using murine genetic models, Kirchner et al. [26] also suggested that GOAT was required to mediate the impact of dietary MCTs on body adiposity. Recent studies have suggested that GOAT might be a therapeutic target against obesity and hyperphagia by inhibiting the GOAT activity [27], in order to decrease the circulating level of acylated ghrelin (which may also be obtained by limiting the availability of its substrate C8:0) [27].

Altogether, these data support contradictory physiological effects of dietary caprylic acid on body weight and food consumption. We therefore wanted to clarify the discrepancy between the historically described beneficial effects of dietary MCFAs including C8:0 on body weight and fat loss and its newly reported effect on food consumption and appetite stimulation *via* ghrelin acylation. The purpose were to (i) define the origin of caprylic acid which is used to acylate ghrelin in the stomach, (ii) study the effect of increasing dietary caprylic acid levels on the C8:0 available in the stomach and other tissues, (iii) analyze the subsequent effect of increasing dietary caprylic acid levels on the concentration of circulating plasma acylated and non-acylated ghrelin, (iv) study the whole effect of increasing dietary caprylic acid levels on dietary consumption, body weight and on other parameters such as adiposity. Here, we showed that dietary C8:0 led to a specific dose-response enrichment of C8:0 in the stomach tissue. However, this increased C8:0 availability did not affect the plasma acylated ghrelin concentration but decreased the plasma unacylated ghrelin concentration.

## Materials and Methods

### Chemicals

Solvents and chemicals were purchased from Sigma-Aldrich (Saint-Quentin Fallavier, France) or Thermo Fisher Scientific (Elancourt, France). Tricaprylin was from TCI Europe (Zwijndrecht, Belgium). Tripalmitin was from Sigma. Rat plasma acylated and unacylated ghrelin enzyme immunoassays kits (EIA) were purchased from SPI-Bio (Montigny Le Bretonneux, France). Kits for plasma glucose, cholesterol and triglycerides were purchased from Bio-mérieux (Lyon, France). Rabbit anti-ghrelin polyclonal antibodies (bs-0467R) used for immunohistochemistry were purchased from Bioss (Massachusetts, USA).

### Diets

Three different lipid mixes (Table 1) were prepared with commercial oils (olive, rapeseed, palm, corn and flaxseed oils) and increasing amounts of caprylic acid (0, 8 and 21% of FAs) in the form of tricaprylin substituted with decreasing amounts of palmitic acid (30, 20 and 7.5% of FAs) in the form of tripalmitin. These lipid mixes were used to prepare diets containing either moderate amounts of fat called MF diets (10% in mass, *i*.*e*. 21% of total energy), or high amounts of fat called HF diets (25% in mass, *i*.*e*. 45% of total energy), at the Unité de Production d’Aliments Expérimentaux (INRA, Jouy en Josas, France). The three MF diets (MF-0, MF-8 and MF-21) also contained 42.2% of starch, 21.1% of sucrose, 19.9% of casein, 1.8% of cellulose, 4.1% of mineral mix and 0.9% of vitamin mix. These MF diets were isoenergetic (423 kcal/100 g) and contained the same amount of total saturated fatty acids (SFAs), caprylic acid representing 0, 1.7 and 4.4% of total energy, respectively. The MF diets were provided *ad libitum* to the rats but also as calorie restricted rations (CR diets) corresponding to 70% of *ad libitum* and thereafter called CR-0, CR-8 and CR-21 diets. Finally, in the three HF diets (HF-0, HF-8 and HF-21) containing 25% in mass of fat, the increase in fat level was counterbalanced with a decrease in starch (32.2%) and sucrose (16.1%). These HF diets were also isoenergetic (498 kcal/100 g), caprylic acid representing 0, 3.6 and 9.5% of total energy, respectively.

**Table 1:** Fat mixture and fatty acid composition (% of FAs) of the experimental lipid blends containing increasing levels of caprylic acid (C8:0).

	Diet-0%	Diet-8%	Diet-21%
**Fat (%)**			
Olive Oil	47.0	48.3	50.0
Rapeseed Oil	0.0	0.0	10.0
Palm Oil	19.0	17.0	0.0
Corn Oil	13.5	13.5	13.3
Flaxseed Oil	5.5	5.5	3.7
Tricaprylin	0.0	10.0	23.0
Tripalmitin	14.7	5.7	0.0
**FA (% of FAs)**			
C8:0	0.0	8.4	20.6
C12:0	0.1	0.1	0.0
C14:0	0.3	0.3	0.0
C16:0	30.0	19.8	7.6
C18:0	3.1	2.9	2.2
C20:0	0.3	0.3	0.3
C22:0	0.0	0.1	0.1
**ΣSFA** ^1^	**33.8**	**31.8**	**30.9**
C16:1 (n-9)	0.0	0.1	0.1
C16:1 (n-7)	0.4	0.4	0.4
C18:1 (n-9)	47.7	49.3	49.9
C18:1 (n-7)	1.7	1.6	1.9
C20:1 (n-9)	0.2	0.2	0.3
**ΣMUFA** ^1^	**50.0**	**51.5**	**52.5**
C18:2 (n-6)	12.7	13.2	13.1
C18:3 (n-3)	3.5	3.5	3.5
**ΣPUFA** ^1^	**50.0**	**51.5**	**52.5**
*C18*:*2/C18*:*3 ratio*	*3*.*7*	*3*.*8*	*3*.*8*

The three different lipid mixes were used for preparing the MF, CR and HF diets.

^1^ΣSFA, ΣMUFA and ΣPUFA correspond respectively to the sum of saturated, mono-unsaturated and poly-unsaturated fatty acids.

### Animals

All protocols complied with the European Union Guideline for animal care and use (2003/35/CEE). The experimental procedure (n°01374.02) was approved by the French Animal Care Committee (Rennes) and the Ministry of Higher Education and Research, in compliance with recommendations of the 2013–118 directive for animal experimentation. Forty five Sprague-Dawley male rats (60 g weight) were purchased from Janvier breeding center (Le Genest Saint Isle, France). Eighteen rats were randomly distributed to 3 groups (n = 6) and fed *ad libitum* with the three MF diets (MF-0, MF-8 and MF-21). As mentioned above, three other groups of rats (n = 3) received the same MF diets restricted to 70% of *ad libitum* and were called CR groups (CR-0, CR-8 and CR-21). The n value of the CR groups was limited to 3 rats due to housing restriction. Finally, three other groups of rats (n = 6) were fed *ad libitum* with the HF diets (HF-0, HF-8 and HF-21). The nine different groups of rats were fed during 6 weeks with the experimental diets. They had free access to water. Rats were housed in groups of two (MF and HF diets) or alone (CR diets) at 19°C-23°C with a 12 h light-dark cycle.

Animal weight and food consumption were measured daily. Blood samples were collected every week starting from the second week of diets (between 8 and 11 am) in the tail vein alternatively on 18 h fasted (2^nd^ and 4^th^ weeks) or fed state (3^rd^ and 5^th^ weeks) in order to measure the plasma acylated and unacylated ghrelin concentrations. For ethical reasons, rats already under chronic calorie restriction were not additionally deprived of food and no results are therefore presented for the CR group under fasted conditions. After 6 weeks of experiment, rats were anesthetized following an overnight fast with an intraperitoneal injection of pentobarbital (140 mg/kg) (Merial, Lyon, France) and blood samples were collected by cardiac punctures. The liver, the brain, the mesenteric and the subcutaneous adipose tissues were removed, weighted, snap-frozen in liquid nitrogen and stored at -80°C. Sections of subcutaneous adipose tissues were directly fixed in formalin for morphometric analysis. The intestine (duodenum, jejunum and ileum) was emptied, rinsed with ice-cold saline solution, snap-frozen and stored at -80°c. The stomach was subsequently removed and treated similarly. Stomach longitudinal sections were collected and fixed in formalin for immunohistology.

### Measurement of the plasma acylated and unacylated ghrelin

Blood samples collected in the tail vein were immediately mixed with EDTA (1 mg/ml plasma) and with the serine protease inhibitor p-hydroxymercuribenzoic acid (PHMB, 0.4 μM final) to protect the acylated ghrelin from enzymatic degradation [28]. The plasma was rapidly separated from the blood cells by centrifugation and acidified by adding HCl (0.1 N final) to preserve ghrelin acylation, as well. Samples were then stored at -80°C for later hormone titration. The acylated and unacylated ghrelin concentrations were assayed with the commercial EIA kits [29] according to the manufacturer’s instructions.

### Analysis of the other plasma parameters

The plasma collected by cardiac punctures at the end of the experiment was separated from the blood cells by centrifugation. Plasma glucose, cholesterol and TG were assayed with commercial enzymatic kits [30] according to the manufacturer’s instructions.

### Lipid extraction and FA analysis

Lipids from liver, stomach, duodenum, jejunum, ileum, epididymal, mesenteric and subcutaneous adipose tissues were extracted using a mixture of dimetoxymethane/methanol (4:1 v/v) after homogenization with an Ultra-Turrax as previously described [31]. Lipids from plasma were extracted with a mixture of hexane/isopropanol (3:2 v/v), after acidification with 1 ml HCl 3 M [32]. Pelargonic acid (C9:0) and margaric acid (C17:0) were added as internal standards in order to quantify MCFAs (with C9:0) and LCFAs (with C17:0). Total lipid extracts were saponified for 30 min at 70°C with 1 ml of 0.5 M NaOH in methanol and methylated with 1ml BF_3_ (14% in methanol) for 15 min at 70°C. To protect the presence of volatile short and medium chain FAs, FA methyl esters (FAMEs) were extracted by adding 1 ml hexane and 5 ml NaCl 0.9% in deionized water [33]. After shaking, the hexane phase was directly analyzed by gas chromatography coupled to mass spectrometry (GC-MS) [34] using an Agilent Technologies 7890N (BiosAnalytic, Toulouse, France) with a split injector (10:1) at 250°C and a bonded silica capillary column (BPX70, 60 m × 0.25 mm, SGE, Villeneuve-St-Georges, France). The column temperature program started at 45°C and stayed at this temperature for 3 min in order to analyze MCFAs. Then, the temperature ramped firstly at 10°C/min to 150°C, then at 1.4°C/min to 220°C and at 40°C/min to 260°C and held at 260°C for 2 min.

### Quantification of mRNA expression by Real-Time PCR

Total RNA was extracted from stomach, hypothalamo-pituitary complex, mesenteric adipose tissue and liver with Trizol. RNA was converted into cDNA with the IScriptcDNA synthesis kit (Bio-Rad) according to the supplier’s instructions. RNA was quantified by real-time qPCR with the SsoFast EvaGreen supermix (Bio-Rad) containing 50 ng of retrotranscripted RNA, 300 nM of Forward primers and 300 nM of Reverse primers and 10 μl SsoFast EvaGreen supermix. The sequences of primers were designed as described in Table 2. Quantitative real-time PCR was done in optical 96-well plates on a CFX 96 real-time PCR Detection System (BioRad) as follows: 30 s at 95°C, 40 cycles of 5 s at 95°C and 5 s at 55°C. The 18S mRNA level was quantified as an endogenous control.

**Table 2:** Primers designed for quantitative RT-PCR analysis of rat genes.

Genes	Preproghrelin (PPG)	Insulin-like growth factor-1 (IGF-1)	Growth hormone secretagogue receptor-1a (GHSR-1a)
Species	rat	rat	rat
GenBank Acc. no.	NM_021669.2	NM_001082479.1	NM_032075.3
Forward primer	TGGCATCAAGCTGTCAGGAG	AGTTCGTGTGTGGACCAAGG	GCCATCTGCTTCCCTCTG
Reverse primer	GGCAGAAGCTGGATGTGAGT	GGTGACGTGGCATTTTCTGT	ATCTGTGCCGTTTTCGTG
Amplicon size bp	171	312	144

Ghrelin O-Acyltransferase (GOAT, MBOAT4) mRNA was quantified by Real-Time PCR with the Taqman Universal PCR Master Mix (Applied Biosystem) containing 40 ng of retrotranscripted RNA, 0.5 μM of primers and 0.25 μM of Taqman probe. Primers and 5’-FAM/TAMRA-3’ Taqman probes specific to GOAT gene were designed (forward primer 5’-gggccaggtacctctttctc-3’; reverse primer 5’-cggccaaagccaggacaccct-3’) and Taqman probe (5’FAM-aaatgagcagagcgtaggga-TAMRA3’). Amplification was performed by an ABI PRISM 7000 (Applied Biosytems) over 40 cycles of 95°C for 15 s and 60°C for 1 min. The *18S* gene expression was quantified as an endogenous control using the 18S RNA Control kit Yakima-Yellow Eclipse Dark Quencher (Eurogentec) and relative gene expression was determined from the cycle thresholds (Ct) using the ∆∆Ct method.

### Determination of adipocyte size (morphometric analysis)

Subcutaneous adipose tissue was fixed with 4% buffered formalin, pH 7.4, during 1 week at 4°C and embedded in paraffin [35,36]. Three 4 μm sections per rat shifted by 150 μm were cut using a microtome LEICA (Milton Keynes, UK) and the sections were mounted onto glass slides and stained in Hemalun-Eosin-Safran (H.E.S). The adipocyte areas were manually traced and analyzed using the NIS Elements software. Adipocyte diameter, area and perimeter were calculated for 5 separate photographs per tissue sections. These data were used to calculate the frequency distribution of adipocyte diameters and the mean diameter.

### Immunohistochemistry

Longitudinal stomach sections were fixed with 4% buffered formalin, pH 7.4, during 24 hours and embedded in paraffin [37]. Paraffin-embedded tissue was cut at 4 μm and slides were mounted onto glass slides. Immunohistochemistry was performed with rabbit polyclonal ghrelin (acylated and unacylated) antibodies, targeting an amino-acid sequence of the ghrelin which does not include the octanoylation site. Immunohistochemical staining was performed on Automated IHC stainer using the LEICA ST502. The signal was counted using the NIS elements software.

### Statistical analysis

Statistical analyses were realized using GraphPad Prism (GraphPad Software Inc., San Diego, CA, USA). Group differences were analyzed by the non-parametric Kruskal-Wallis test with the Dunn’s post-tests or with non-parametric Mann-Whitney test to compare the amount of C8:0 between two sections of the stomach. A two-way ANOVA (not repeated measures) was used to analyze the effect of the diet and the localization in the stomach (fundus or corpus) on the C8:0 availability, and the effect of the tricaprylin on the frequency distribution of adipocyte diameter. Body weight gain was analyzed using the two-way ANOVA with repeated measures. All Data are expressed as the mean ± SEM and for all tests, P<0.05 was considered as statistically significant.

## Results

### C8:0 availability in the stomach, intestine and other tissues

**Fig 1:**
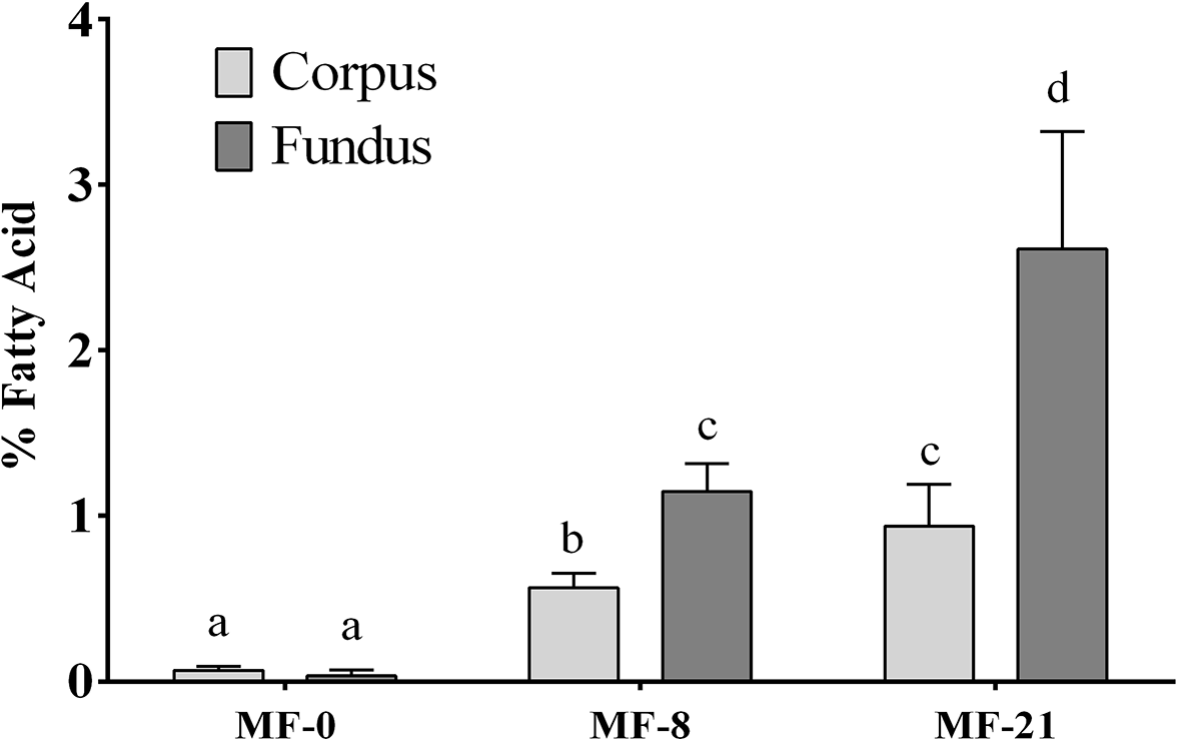
Effect of dietary tricaprylin level on C8:0 availability in the stomach tissue in the MF groups. The stomach tissue was dissected and the fatty acids of the fundus and corpus were analyzed. The C8:0 available in the different parts of the stomach is expressed as mean ± SEM in % of FAs in the tissues. The effect of the diet and the localization in the stomach tissue was analyzed using two-way ANOVA: (i) effect of the diet on C8:0 availability, P<0.0001; (ii) effect of the part of the stomach tissue studied on C8:0 availability, P = 0.0092; (iii) interaction between the two factors, P = 0.0387.

The effect of dietary C8:0 (0, 8 and 21% of FAs) was studied in rats subjected to three different treatments: *ad libitum* Moderate Fat (MF) diet, Calorie Restricted (CR) diet (70% of MF *ad libitum* energy intake) and *ad libitum* High Fat (HF) diet. The composition of total FAs was analyzed in several tissues to determine the metabolic fate of dietary C8:0. The amount of C8:0 expressed as % of FAs in the tissues of MF rats is shown in Table 3. Regardless its amount in the diets, no C8:0 was detected in the plasma and in the liver of rats consuming the MF diets. In adipose tissues, the amount of C8:0 significantly increased with the level of dietary C8:0 but only reached about 0.3% of total FAs in the MF-21 group. In the stomach tissue, a clear dose-response enrichment of C8:0 was observed. Interestingly, even if no C8:0 was present in the MF-0 diet, a very low but detectable amount of this specific FA was found in the stomach tissue. In the stomach of the MF-8 and MF-21 rats, the percentage of C8:0 (in % of FAs) was 3 to 4-fold greater than in adipose tissues of the same animals. Although lower than in the stomach, a dose-dependent increase in C8:0 concentration was also observed in the first sections of the intestine (duodenum and jejunum). Finally, the FA analysis of the two parts of the stomach (fundus and corpus) was realized in the MF groups (Fig 1) and showed again a dose-dependent increase in C8:0, the level of C8:0 being significantly higher in the fundus than in the corpus.

**Table 3:** Effect of dietary tricaprylin (0, 8, 21% of FAs) in the MF groups on the C8:0 availability in different tissues.

C8:0 in % FA				
Diets	MF-0	MF-8	MF-21	K-W test
Plasma	n.d.^1^	n.d.	n.d.	n.s.^2^
Liver	n.d.	n.d.	n.d.	n.s.
Mesenteric adipose tissue	n.d.	0.15 ± 0.00	0.25 ± 0.01	*
Subcutaneous adipose tissue	n.d.	0.15 ± 0.01	0.26 ± 0.02	
Epididymal adipose tissue	n.d.	0.19 ± 0.01	0.32 ± 0.01	*
Stomach	0.07 ± 0.03	0.48 ± 0.07	1.22 ± 0.25	**
Duodenum	n.d.	0.14 ± 0.01	0.43 ± 0.09	*
Jejunum	n.d.	0.12 ± 0.03	0.30 ± 0.12	n.s.
Ileum	n.d.	n.d.	n.d.	n.s.

Results are expressed as mean ± SEM in % of FAs in the tissues. Kruskal-Wallis test (K-W test)

*P<0.05

**P<0.01.

^1^ not detected.

^2^ statistically not significant.

Concerning palmitic acid (S1 Table), the higher level of dietary C16:0 in MF-0 rats led to a significant increase in the % of C16:0 only in mesenteric and subcutaneous adipose tissues, compared to MF-8 and MF-21 rats. The lower intake in palmitic acid in MF-8 and 21 diets was likely compensated by an increased endogenous synthesis of this FA.

### Plasma acylated and unacylated ghrelin

#### Under fed conditions

**Fig 2:**
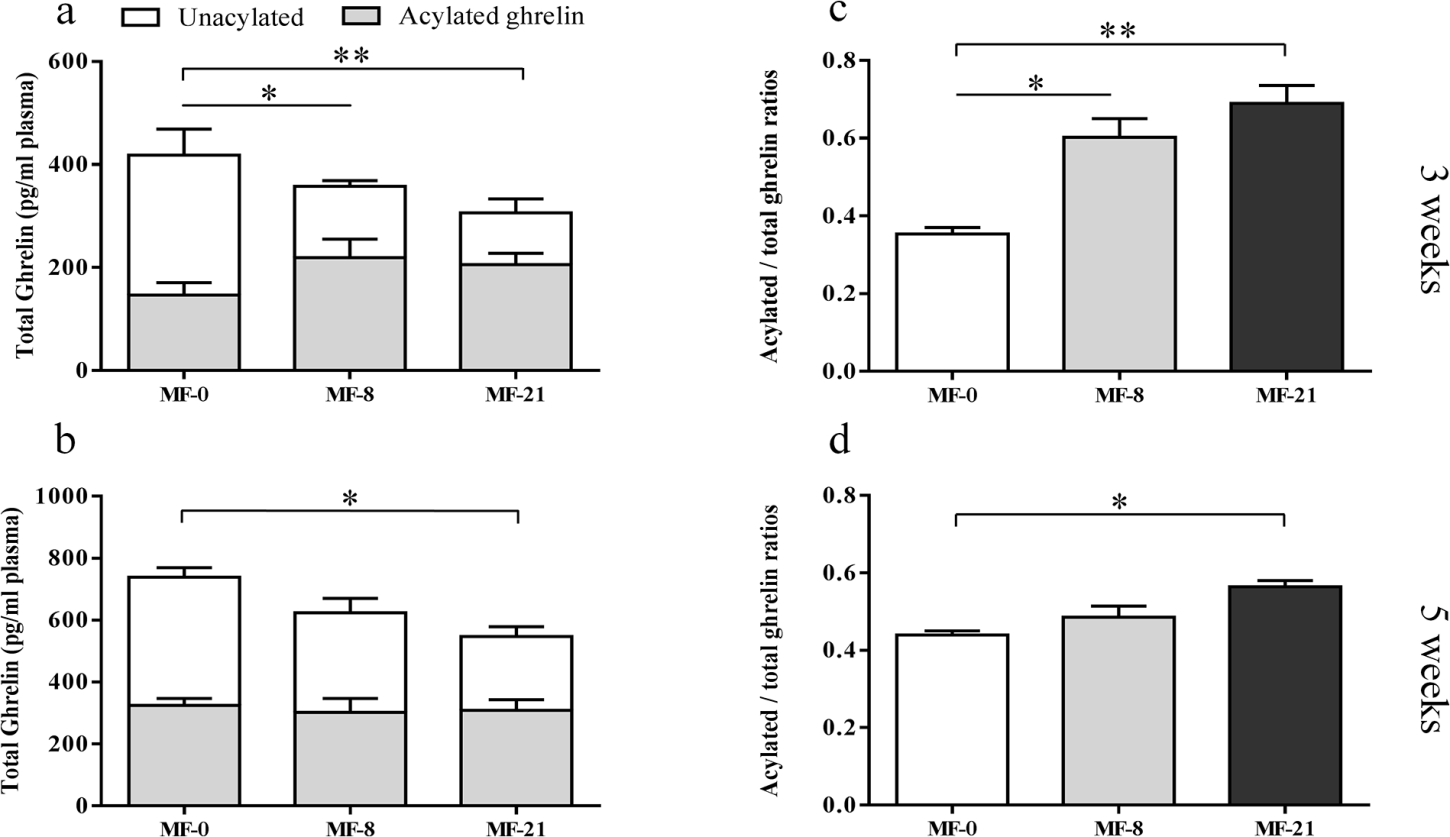
Effect of dietary tricaprylin level on the plasma acylated and unacylated ghrelin concentrations, in the MF groups (fed state). (**a**) Acylated and unacylated ghrelin concentrations after 3 weeks of diet. Kruskal-Wallis test (K-W): (i) acylated ghrelin, P = 0.44; (ii) unacylated ghrelin, P = 0.0018, Dunn’s post tests, *P<0.05, **P<0.01; (iii) total ghrelin, P = 0.5824. (**b**) Acylated and unacylated ghrelin concentrations after 5 weeks of diet. K-W test: (i) acylated ghrelin, P = 0.56; (ii) unacylated ghrelin, P = 0.029, Dunn’s post tests, *P<0.05; (iii) total ghrelin, P = 0.127. (**c**) Acylated/total ghrelin ratios after 3 weeks of diet. K-W test: P = 0.006, Dunn’s post tests, *P<0.05, **P<0.01. (**d**) Acylated/total ghrelin ratios after 5 weeks of diet. K-W test: P = 0.0103, Dunn’s post tests, *P<0.05.

The plasma acylated and unacylated ghrelin concentrations were measured in the blood collected on rats under fed conditions after 3 and 5 weeks on the experimental diets (Fig 2). The plasma total ghrelin was calculated by adding the acylated and unacylated ghrelin concentrations. After 3 weeks (Fig 2A) and 5 weeks (Fig 2B), the acylated ghrelin concentration was not modified by the dietary C8:0 levels. Conversely the concentration of unacylated ghrelin was significantly lower in the MF-8 and MF-21 groups than in the control group after 3 weeks (Fig 2A) and significantly lower in the MF-21 group than in the control group after 5 weeks (Fig 2B). Due to the lower levels of unacylated ghrelin, the total ghrelin concentration was also decreased when C8:0 was present in the diets. As a consequence, the acylated to total ghrelin ratios were significantly higher (Fig 2C) in the MF-8 and MF-21 groups than in the control group after 3 weeks and significantly higher in the MF-21 group compared with the control group with intermediate level for the MF-8 group after 5 weeks (Fig 2D).

Plasma acylated and unacylated ghrelin concentrations were similarly measured in the blood collected on fed rats of the HF groups (S1 Fig). Once again, no effect of dietary C8:0 was shown on the acylated ghrelin concentrations but a significantly lower level of unacylated ghrelin concentrations was detected in the HF-21 group than the HF-0 group. In the CR groups, the C8:0 dietary level did not modify significantly the concentration of acylated and unacylated ghrelin whatever the diet duration (S2 Fig).

#### Under fasted conditions

**Fig 3:**
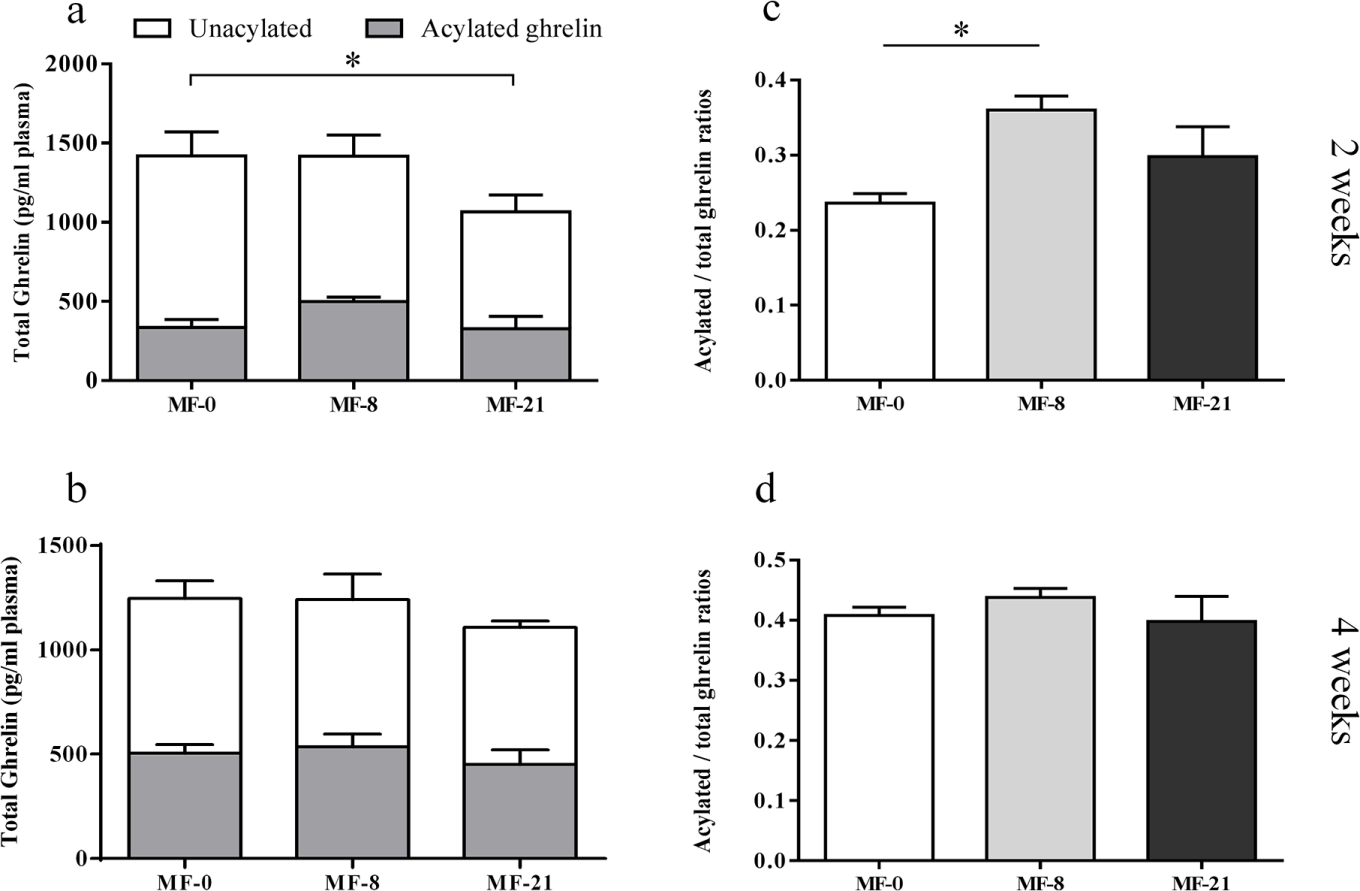
Effect of dietary tricaprylin level on the acylated and unacylated ghrelin concentrations, in the MF groups (fasted state). (**a**) Acylated and unacylated ghrelin concentrations after 2 weeks of diet. Kruskal-Wallis test (K-W): (i) acylated ghrelin, P = 0.0837; (ii) unacylated ghrelin, P = 0.038, Dunn’s post tests, *P<0.05; (iii) total ghrelin, P = 0.2345. (**b**) Acylated and unacylated ghrelin concentrations after 4 weeks of diet. K-W test: (i) acylated ghrelin, P = 0.4585; (ii) unacylated ghrelin, P = 0.5643; (iii) total ghrelin, P = 0.65. (**c**) Acylated/total ghrelin ratios after 2 weeks of diet. K-W test: P = 0.0181, Dunn’s post tests, *P<0.05. (**d**) Acylated/total ghrelin ratios after 4 weeks of diet. K-W test: P = 0.3635.

The plasma acylated and unacylated ghrelin concentrations were also measured in the blood collected on rats under fasted conditions after 2 and 4 weeks on the experimental diets (Fig 3). Fig 3A and 3B show that the concentrations of both acylated and unacylated ghrelin were significantly higher when MF rats were under fasted conditions than under fed conditions (Fig 2). Whatever the diet duration, the plasma acylated ghrelin concentration was not significantly modified by the consumption of C8:0. After 2 weeks, the unacylated ghrelin concentration was significantly lower in the MF-21 than in MF-0 group (Fig 3A), as already shown under fed conditions. A similar but not significant downward trend was shown after 4 weeks (Fig 3B). Consequently, a significant increase in the acylated/total ghrelin ratio was shown after 2 weeks between the MF-8 and MF-0 (Fig 3C), but this effect disappeared gradually after longer dietary periods (Fig 3D), as also shown previously under fed conditions.

Concerning the rats of the HF groups under fasted conditions (S3 Fig) no significant effect of the C8:0 dietary level was shown after 2 and 4 weeks on acylated or unacylated ghrelin concentrations.

### Stomach PPG mRNA level and ghrelin peptide expression

**Fig 4:**
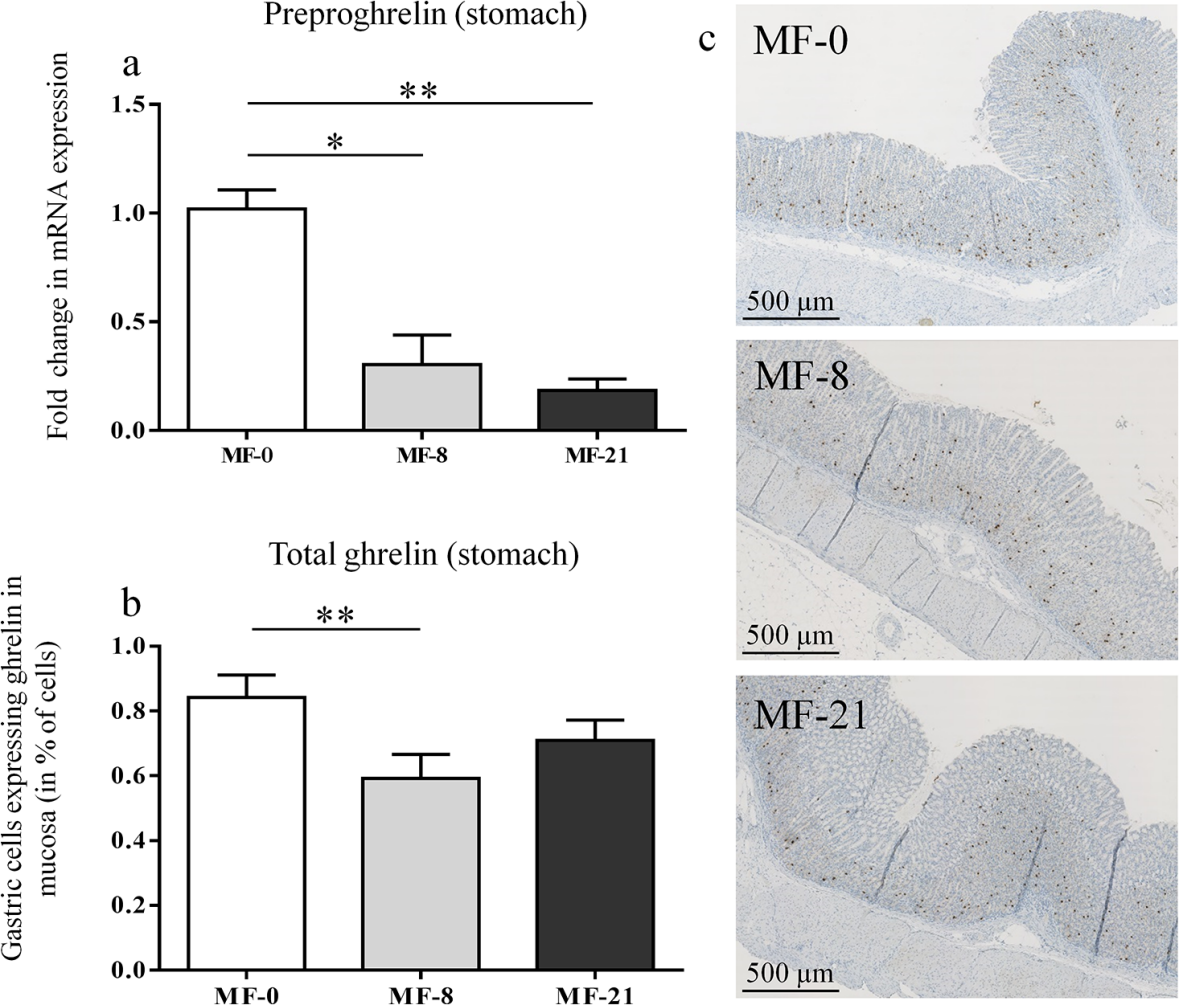
Effect of tricaprylin on total ghrelin expression in the MF groups. (**a**) Preproghrelin mRNA level (in fold change of the control MF-0) in the stomach tissue. Kruskal-Wallis (K-W) test: P = 0.004, Dunn’s post tests, *P<0.05, **P<0.01. (**b**) Quantification of total ghrelin staining in the gastric mucosa of the corpus. K-W test: P = 0.017, Dunn’s post tests, **P<0.01. (**c**) Ghrelin staining in gastric mucosa of MF-0, MF-8 and MF-21 groups.

The stomach mRNA level of preproghrelin (PPG) gene was measured in the MF groups (Fig 4A) showing a linear decrease when dietary C8:0 increased. The expression level of ghrelin peptide was quantified by immunochemistry in longitudinal sections of gastric mucosa for the MF groups (Fig 4B and 4C) and the signal was measured in the corpus. The percent of cells expressing ghrelin in the corpus was higher in the MF-0 group (0.9% of total cell in the mucosa) than in MF-8 or MF-21 groups (0.6% and 0.7% respectively) (P<0.01). The stomach mRNA level of ghrelin O-acyltransferase (GOAT) gene was also measured in the MF groups (S4 Fig) showing no significant effect of dietary C8:0 on the enzyme expression.

### Analysis of parameters putatively controlled by plasma ghrelin

#### Body weight and dietary consumption

**Fig 5:**
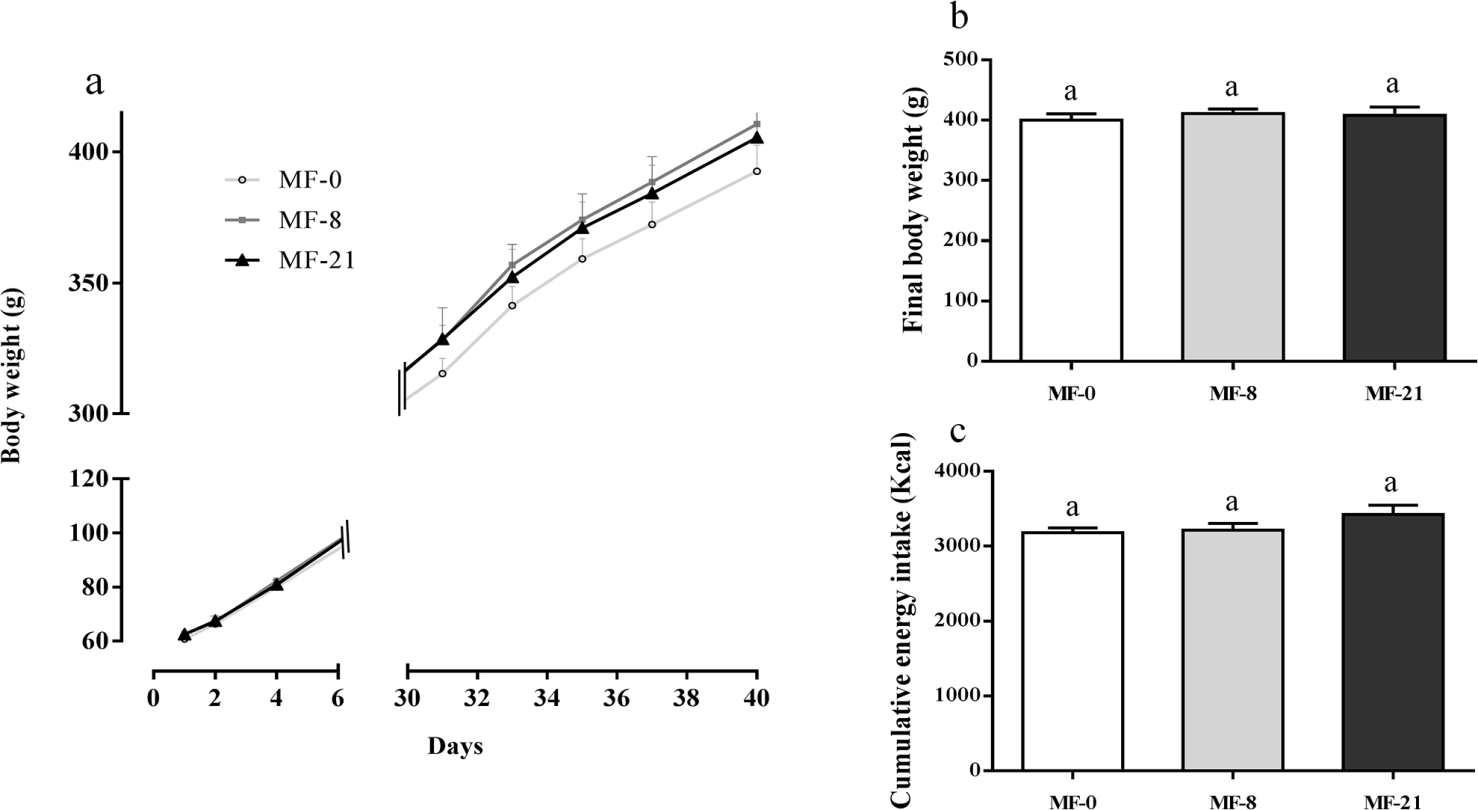
Effect of tricaprylin on body weight gain and food intake in the MF groups. (**a**) Body weight gain in gram. Two-way repeated measurement ANOVA: effect of the diet, P = 0.5275. (**b**) Final body weight in gram; Kruskal-Wallis (K-W) test: P = 0.9098. (**c**) Cumulative energy intakes in kcal. K-W test: P = 0.3393. Results are expressed as mean ± SEM.

In the MF groups, no significant differences on body weight gain, final body weight and cumulative energy intakes were observed (Fig 5A, 5B and 5C). In the CR-groups and HF groups (S5 Fig) no effect of the level of C8:0 was shown on the weight gain and energy intake.

#### Fat mass and adiposity

**Fig 6:**
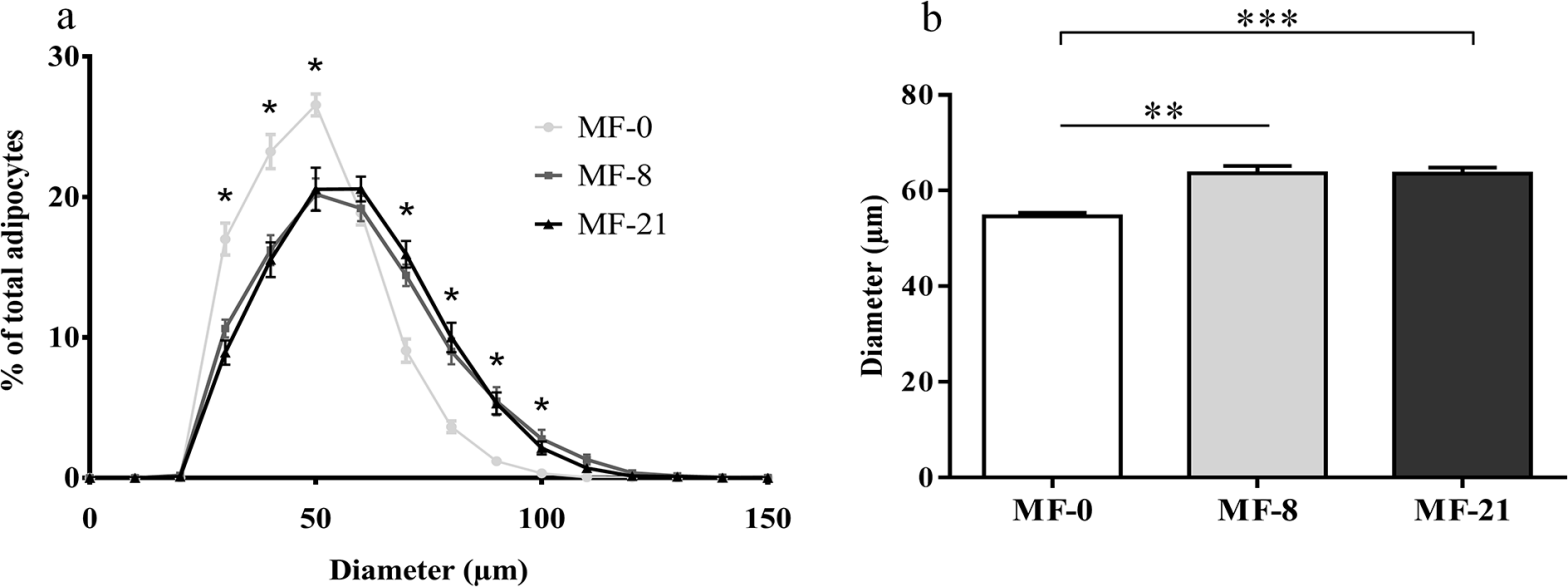
Effect of tricaprylin in the MF groups on adiposity. (**a**) Frequency distribution of adipocyte diameter (μm). (**b**) Adipocyte diameter (μm), K-W test, **P<0.01, Dunn’s ***P<0.001. Results are expressed as mean ± SEM.

Because adipose tissues are target organs for ghrelin [38] and are also influenced by consumption of FAs, the impact of the diets on fat pad masses and on histological parameters of subcutaneous adipose tissues was studied. Fat pad masses were measured for several adipose tissues (mesenteric, epididymal, subcutaneous, retroperitoneal and perirenal) in rats fed with the MF, CR and HF diets and no significant differences were observed (S6 Fig). In the subcutaneous adipose tissue of the MF rats, Fig 6 shows that rats consuming both the MF-8 and -21 diets exhibited an increased frequency of larger adipocytes compared with the control group. As a consequence, the mean adipocyte size (Fig 6B) in the subcutaneous fat of rats fed with both MF-8 and MF-21 (respectively 63 ± 2 *μm* and 63 ± 2 *μm*) was significantly larger than in rats fed with the control diet (54 ± 1 *μm*) (P<0.0001).

#### Plasma parameters

The results obtained by measuring TG, total CH and glucose in plasma collected at the sacrifice are presented in S2 Table. In the MF groups, no effect of increasing dietary amounts of C8:0 was shown on these plasma parameters. The CR and HF rats also displayed the same triglyceridemia and glycemia between groups receiving dietary C8:0 and control groups.

#### Liver IGF-1 and pituitary GHSR-1a mRNA levels

**Fig 7:**
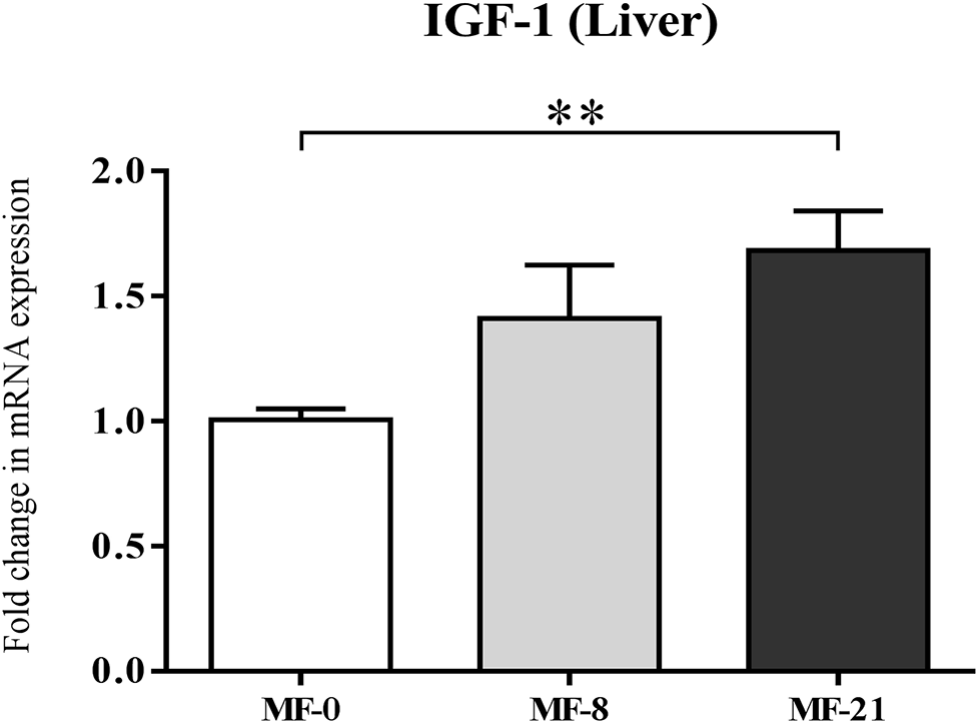
Effect of C8:0 in the MF groups on liver IGF-1 expression. IGF-1 mRNA level (in fold change). K-W test, P = 0.009, Dunn’s post tests, *P<0.05. Results are expressed as mean ± SEM.

The liver mRNA level of the IGF-1 gene (Fig 7) was analyzed because it is directly influenced by the GH secretion, itself regulated by the acylated ghrelin *via* its binding to GHSR-1a receptor [39]. The expression was higher in both MF-8 and MF-21 groups than in the control group, with a significant effect between MF-21 and MF-0 rats. The analysis of the mRNA level of GHSR-1a gene in the hypothalamo-pituitary complex did not show any difference of expression between the MF rats (data not shown).

## Discussion

The discovery of ghrelin as the endogenous ligand of GHSR-1a receptor [17] and the description of its GOAT-catalyzed essential post-translational octanoylation [23,24] brought to light a new issue which is to understand the physiological functions regulated by dietary C8:0. The present study aimed at describing the effect of moderate to high dietary enrichment in caprylic acid on several physiological parameters which may be either directly regulated by C8:0, since MCFAs have specific metabolic properties, or indirectly by its ability to acylate the ghrelin. The animals received three dietary caprylic acid levels (0, 8 and 21% of FAs) in three different nutritional conditions (moderate fat, caloric restriction and high fat). The rationale for such an experimental design was to exacerbate the potential effects of dietary caprylic acid in different nutritional conditions. Indeed, it has been shown in Goat(-/-) mice that the GOAT-ghrelin axis helped to regulate glucose homeostasis in case of prolonged calorie restriction [22,40]. In addition, it is known that the nutritional conditions influence the concentrations of acylated and unacylated ghrelin [26,41]. Except the substitution with decreasing levels of the long chain saturated C16:0, the diet FA compositions were set to values achievable in human nutrition when expressed as % energy [42].

We first investigated the effect of the three dietary caprylic acid levels on the stomach C8:0 availability to further correlate them with the plasma acylated ghrelin concentrations. C8:0 was not detected in the liver or in the plasma (Table 3) after an 18h fast, confirming that it had been rapidly taken up and catabolized by the liver [43], even in rats fed during 6 weeks with high amounts of caprylic acid. Additionally, only a very low storage of this FA was shown in different adipose tissues even with the higher C8:0 dietary level (Table 3), which is also consistent with the described portal vein transport of MCFAs as free fatty acids [7]. Finally, dietary C8:0 increased the C8:0 level in the stomach tissue more than in all other tissues (Table 3). Moreover, the C8:0 amount was 2- to 3-fold higher in the fundus than in the corpus of both MF-8 and -21 groups (Fig 1). These results are therefore consistent with an early preduodenal hydrolysis of tricaprylin and direct absorption of C8:0 at the gastric level, starting in the first part of the stomach. The digestion of dietary fat is a complex and dynamic process which starts in the upper gastrointestinal tract under the action of preduodenal lipase (gastric lipase in humans or its homologous lingual lipase in rats) and is completed in the small intestine by pancreatic lipase. Although the existence and functional importance of a preduodenal lipase was disputed in the past [4], it is now described as contributing to 15–20% of the whole lipolysis process [44]. This preduodenal lipase may play two important roles in triggering the action of pancreatic lipase [45] and driving the early release of short and medium chain FAs that can therefore be directly absorbed in the stomach [5], which is consistent with our results (Table 3). The amounts of MCFAs absorbed by the gastric mucosa, the mechanism of this absorption [46] and the metabolic fate of C8:0 which could be used to acylate proghrelin are still unknown. Since the maximum C8:0 stomach content of the rats fed with the highest enriched diet only reached 1.2% of the total FAs (Table 3), our results suggested either that this absorption was limited or that the absorbed gastric caprylic acid was rapidly catabolized. Interestingly, in the control group receiving no C8:0, a very low but detectable amount of C8:0 (0.07% of FAs) was found in the stomach (Table 3). This suggests a possible endogenous synthesis which has been described for instance in rat liver for C14:0 but not yet for C8:0 [47]. Thus, the present work demonstrates that dietary C8:0 provided the stomach cells (especially the fundus cells) with caprylic acid.

The next issue was to determine if this higher available stomach C8:0 level influenced the stomach total ghrelin expression and increased the concentration of circulating acylated ghrelin, after the action of the gastric GOAT enzyme. Indeed, Nishi et al. detected heptanoylghrelin [25] or decanoylghrelin [48] in the stomachs of mice fed with triheptanoin or tricaprin. This suggests that ingested MCFAs were directly utilized for the ghrelin acylation. In ruminants, the ingestion of MCFAs during 2 weeks by lactating dairy cows also increased plasma acylated ghrelin concentrations [49]. In the present study, the stomach preproghrelin mRNA levels (Fig 4A) and the total ghrelin peptide (acylated and unacylated forms) expression, quantified by immunohistochemistry in the stomach (Fig 4B and 4C), displayed a linear decrease when dietary C8:0 increased. The plasma acylated ghrelin concentration was stable regardless the dietary C8:0 levels in fed (Fig 2A and 2B) and fasted (Fig 3A and 3B) MF rats. A similar stability was shown in fed (S1A and S1B Fig) and fasted (S3A and S3B Fig) HF rats. In contrast, a reproducible significant decrease in the plasma unacylated ghrelin concentration was shown in fed (Fig 2A and 2B) and fasted (Fig 3A and 3B) MF rats with the increase in dietary C8:0. This decrease was also shown in fed HF rats (S1A and S1B Fig), but not in fed CR rats (S2A and S2B Fig) which may be explained by the rapid food consumption of some of the CR animals (fed at 2 p.m. the day before blood collection) that made the nutritional status (fed or fasted) of the animals more random.

The decrease in unacylated ghrelin led to a reproducible increase in the acylated/total ghrelin ratio in fed and fasted MF rats (Fig 2C and 2D) and in fasted HF rats (S3C and S3D Fig). A similar although not significant increase was shown in fed HF rats (S1C and S1D Fig). As shown by these ratios, acylated ghrelin accounted for 30–60% of the total circulating ghrelin with the highest ratios in the plasma of rats fed with 21% C8:0. Although circulating acylated ghrelin was generally described as 10% of the total ghrelin in humans [50], acylated ghrelin concentration has been experimentally underestimated due to its quick degradation [51]. It seems that acylated ghrelin could actually represent around 30 to 50% of the total ghrelin in humans and in rodents [25], which would be consistent with our results.

These results may appear to be different from those obtained from previous studies showing that the ingestion by mice of free MCFAs or MCTs increased the stomach acylated ghrelin whereas the total amount of ghrelin remained unchanged [25]. However, there are still conflicting results regarding the effects of dietary lipids on ghrelin response. Initially, it was proposed that a fat-rich diet decreases plasma total ghrelin levels [52], but more recently it has been established that the suppression of ghrelin by lipids is relatively weak [53]. The maturation and secretion of stomach ghrelin is indeed a complex process regulated by different parameters such as dietary LCFAs and MCFAs and by fat sensing receptor (G protein-coupled receptor 120, free fatty acid receptor 1) [54]. The circulating ghrelin is additionally submitted to clearance and rapid de-acylation or degradation. For instance, a ghrelin deacylation enzyme (acyl-protein thioesterase-1, APT1) has recently been described, that can des-acylate ghrelin in the plasma [55]. Moreover, acylated ghrelin has been shown to form a complex with larger proteins like immunoglobulins (Ghrelin-reactive IgG) [56] that protects the acylated form from degradation. Some studies assaying total ghrelin in plasma have also reported lower levels in obese subjects, due to lower levels of unacylated ghrelin, whereas acylated ghrelin remained stable, suggesting a specific decreased degradation of acylated ghrelin in obese [56]. In humans, it has also been shown that the GOAT enzyme was present in the blood, which could modify the balance between de-acylation and re-acylation [57]. In a study on the role of the gustatory G-protein in the sensing of FAs for the octanoylation of ghrelin, ingested MCFAs increased stomach acylated ghrelin but did not change the plasma ghrelin concentration [58]. For all these reasons, the concentration of both the acylated and unacylated plasma ghrelin may not simply reflect the stomach concentration [59]. In the present study, we observed decreased unacylated ghrelin and stable acylated ghrelin concentrations in the plasma and decreased total ghrelin in the stomach, with tricaprylin consumption. We can therefore speculate that the secretion of ghrelin from the stomach to the plasma remained unchanged with tricaprylin consumption. The stable levels of stomach GOAT mRNA (S4 Fig) in the MF animals also suggest that C8:0 had no effect on ghrelin octanoylation but the activity of the enzyme was not assessed. Interestingly, high levels of acylated ghrelin were measured in the MF-0 rats receiving no dietary C8:0. Thus, the low stomach C8:0 level observed in the MF-0 group (Table 3) could be enough to supply the octanoyl-CoA co-substrate used to acylate the proghrelin. Some studies suggested that the availability of MCFAs are rate-limiting for the acylation and activation of ghrelin [60] but our results showed stable concentrations of plasma acylated ghrelin whatever the dietary C8:0 levels and decreasing concentrations of its corresponding unacylated form.

These results raised the question of the physiological impact of the increased acylated/total ghrelin ratio resulting from stable plasma acylated ghrelin and decreased unacylated ghrelin. The acylated ghrelin is best known for its orexigenic actions in the central nervous system, involved in the regulation of food intake and thereby in weight control [61]. In the present study, we did not observe any effect of dietary C8:0 on body weight gain and food intake levels in the MF groups (Fig 5). The rats fed with the HF diets (S5B, S5D and S5F Fig) displayed the same weight gain, final body weight and cumulative energy intake than rats fed with the MF diets. Even fed ad libitum with the high fat diets, these rats have controlled their food intake at a similar level than the MF rats. This may explain why the plasma ghrelin level in HF groups was unchanged compared with MF groups. In the CR groups (S4A, S4C and S4E Fig), the same weight gain and final body weight were observed, but with a trend to decreased final body weight with MF-21 diet consumption. These results are consistent with the stable concentrations of plasma acylated ghrelin and suggest the absence of physiological effects of the increased acylated/total ghrelin ratios. Indeed an intracerebral acylated ghrelin administration increased body weight by stimulating food intake and by inhibiting energy expenditure and fat catabolism [62]. However, ghrelin-null mice did not exhibit altered food intake nor altered expression of hypothalamic neuropeptides involved in the regulation of appetite. This explains why the essentiality of endogenous ghrelin in the regulation of food intake now appears controversial [63]. In addition, when related to dietary lipids and especially dietary MCFAs, the importance of the so-called GOAT-ghrelin-GHSR-1a axis on these parameters is unclear. Nishi et al [48] found no effect on body weight and food intake after feeding mice during two weeks with diets enriched in tricaprylin or tricaprin compared to control diets. However, feeding GOAT-null mice with MCT-enriched diets during 8 weeks resulted in significantly lower body weight compared with control mice [26]. By contrast, the use of MCT-enriched diets in the limitation of adiposity has been somewhat successful in humans [14,15,64,65] and in rats, when substituted for LCFAs in excess [11,12,66]. A systematic review of the literature concluded that MCTs had effectively a short-term reducing effect on weight gain in rats but that this effect disappeared in the long term studies [67], probably due to metabolic adaptation to this diet. In the present study, because dietary C8:0 was shown to be rapidly catabolized, the energy expenditure may have been increased. This may in turn have had an additional inverse effect on ghrelin which is a key component of the regulation of energy balance.

No effects of the dietary C8:0 levels and increasing acylated/total ghrelin ratios were neither shown on the plasma glucose and circulating lipids (S1 Table) in the MF, HF or CR groups. Acylated ghrelin was previously described as a modulator of glucose homeostasis [68], but the implication of the GOAT-ghrelin system was recently shown to be unessential for the maintenance of euglycemia during a prolonged calorie restriction [69].

Finally, since little or no effects of the increased acylated/total ghrelin ratio or decreased unacylated ghrelin were shown on the previously described physiological parameters, we wondered if other parameters involved in the ghrelin-GOAT system regulation could be modulated. Since the acylated ghrelin has a major role to facilitate GH secretion [70], we therefore measured the liver insulin-like growth factor-1 (IGF-1) mRNA levels (Fig 7) in order to obtain an assessment of the average amount of GH being produced. The expression of IGF-1 was significantly higher in the MF-21 rats than in the control group. Because plasma acyl ghrelin was constant in our study, we could hypothesize that the increased ratio of acylated/total ghrelin may have led to the activation of IGF-1 transcription via the GH releasing activity of GHSR-1a. Several studies have shown that unacylated ghrelin may act as a competitive inhibitor of acylated ghrelin for the GHSR-1a receptor [71,72]. Therefore, the decrease in plasma unacylated ghrelin may increase the binding of stable acylated ghrelin to its receptor.

In addition, the animals consuming tricaprylin displayed an increase in the mean diameter of adipocytes in subcutaneous adipose tissue with no increase in the fat mass (Fig 6). This result can be explained either by a lower differentiation of preadipocytes into adipocytes leading to hypoplasia and/or by a hypertrophy of adipocytes. The link between MCFAs and adiposity is unclear since they decreased lipid accumulation in mouse 3T3-L1 preadipocytes [73] but not in human adipocytes [74]. In the present study, it seems difficult to relate the increase in adipocyte diameter given the low presence of C8:0 in adipose tissue. Concerning now the effects of acylated and/or unacylated ghrelin on adipogenesis, bibliographic data are also conflicting [75]. The action of ghrelin on adipogenesis may first be independent on its binding with the Growth Hormone Secretagogue Receptor 1a (GHSR-1a), as demonstrated in several studies [76,77], and we can hypothesize in the present study that the decrease in plasma total ghrelin with tricaprylin consumption led to a decreased differentiation of preadipocytes or increased hypertrophy of adipocytes. The action of ghrelin on adipogenesis could secondly be dependent on the GHSR-1a, and we can then hypothesize that the increased plasma acylated/total ghrelin ratio with C8:0 consumption decreased the differentiation of adipocytes.

To conclude, daily feeding with dietary C8:0 increased the C8:0 level in the stomach cells more than all the other tissues and affected the plasma acylated/total ghrelin ratio by decreasing the concentration of unacylated ghrelin. This increased ratio was however not associated with increased body weight or dysregulation of food consumption, which were previously associated with the ghrelin-GOAT-GHSR-1a axis. In western countries, total dietary MCFAs represent less than 2% of total dietary energy including 1 to 2% of caprylic acid in milk fat. In terms of dietary recommendations, these results do not give any argument to limit the current consumption of foods containing caprylic acid [78]. Further studies are however needed to clarify the dose effect of caprylic acid on adipocyte differentiation.

## Supporting Information

S1 Fig Effect of tricaprylin on acylated and unacylated ghrelin concentrations in HF groups (fed state). (**a**) Acylated and unacylated ghrelin concentrations after 3 weeks of diet. Kruskal-Wallis test (K-W): (i) acylated ghrelin, P = 0.201; (ii) unacylated ghrelin, P = 0.04, Dunn’s post tests, *P<0.05; (iii) total ghrelin, P = 0.033, Dunn’s post tests, ^$^P<0.05. (**b**) Acylated and unacylated ghrelin concentrations after 5 weeks of diet. K-W test: (i) acylated ghrelin, P = 0.304; (ii) unacylated ghrelin, P = 0.023; Dunn’s post tests, *P<0.05; (iii) total ghrelin, P = 0.2938. (**c**) Acylated/total ghrelin ratios after 3 weeks of diet. K-W test: P = 0.0669. (**d**) Acylated/total ghrelin ratios after 5 weeks of diet. K-W test: P = 0.0653.
(TIF)

S2 Fig Effect of tricaprylin on acylated and unacylated ghrelin concentrations in CR groups (fed state). (**a**) Acylated and unacylated ghrelin concentrations after 3 weeks; Kruskal-Wallis test (K-W): (i) acylated ghrelin, P = 0.0714; (ii) unacylated ghrelin, P = 0.1321; (iii) total ghrelin, P = 0.1321. (**b**) Acylated and unacylated ghrelin concentrations after 5 weeks; K-W test: (i) acylated ghrelin, P = 0.9929; (ii) unacylated ghrelin, P = 0.6286; (iii) total ghrelin, P = 0.9929. (**c**) Acylated/total ghrelin ratios after 3 weeks; K-W test, P = 0.0250, Dunn’s post tests, *P<0.05. (**d**) Acylated/total ghrelin ratios after 5 weeks; K-W test, P = 0.2071.
(TIF)

S3 Fig Effect of tricaprylin on acylated and unacylated ghrelin concentrations in HF groups (fasted state). (**a**) Acylated and unacylated ghrelin concentrations after 2 weeks; Kruskal-Wallis test (K-W): (i) acylated ghrelin, P = 0.0958; (ii) unacylated ghrelin, P = 0.5380; (iii) total ghrelin, P = 0.3532. (**b**) Acylated and unacylated ghrelin concentrations after 4 weeks; K-W test: (i) acylated ghrelin, P = 0.6808; (ii) unacylated ghrelin, P = 0.8580; (iii) total ghrelin, P = 0.9826. (**c**) Acylated/total ghrelin ratios after 2 weeks; K-W test, P = 0.0076, Dunn’s post tests, *P<0.05, **P<0.01. (**d**) Acylated/total ghrelin ratios after 4 weeks, K-W test, P = 0.2248.
(TIF)

S4 Fig Effect of C8:0 in the MF groups on stomach GOAT (MBOAT4) expression. GOAT mRNA level (in fold change). K-W test, P>0.05. Results are expressed as mean ± SEM.
(TIF)

S5 Fig Effect of tricaprylin on body weight gain and food intake in CR groups (70% of *ad libitum*) and HF groups. (**a**) Body weight gain (in gram) in CR groups. Two-way repeated measurement ANOVA: effect of the diet, P = 0.0662. (**b**) Body weight gain (in gram) in HF diets. Two-way repeated measurement ANOVA: effect of the diet, P = 0.5743. (**c**) Final Body weight (in gram) after 6 weeks on CR diets. Kruskal-Wallis test (K-W): P = 0.0714. (**d**) Final Body weight (g) after 6 weeks on HF diets. K-W test: P = 0.5097 (**e**) Cumulative energy intake after 6 weeks of CR diets (in Kcal); SEM is equal to zero because the rats ate their whole daily food ration; (**f**) Cumulative energy intake after 6 weeks on HF diets (in Kcal). K-W test: P = 0.9286 Results expressed as mean ± SEM.
(TIF)

S6 Fig Effect of tricaprylin on body fat percentage in MF, CR and HF groups. Masses of mesenteric, epididymal, subcutaneous and retroperitoneal relate to total body weight. K-W test: MF groups, P = 0.9384; HF groups, P = 0.6737; CR groups, P = 0.096.
(TIF)

S1 Table Effect of the diets in the MF groups on the C16:0 availability in different tissues. Results expressed as mean ± SEM.
(TIF)

S2 Table Effect of tricaprylin on triglyceridemia, cholesterolemia and glycemia in MF, CR and HF groups. Results expressed as mean ± SEM.
(TIF)
