# Supplementary figures and images for: Dietary Caprylic Acid (C8:0) Does Not Increase Plasma Acylated Ghrelin but Decreases Plasma Unacylated Ghrelin in the Rat
Source: PLoS One. 2015 Jul 21;10(7):e0133600. doi: 10.1371/journal.pone.0133600 (PMC4509905; doi:10.1371/journal.pone.0133600)

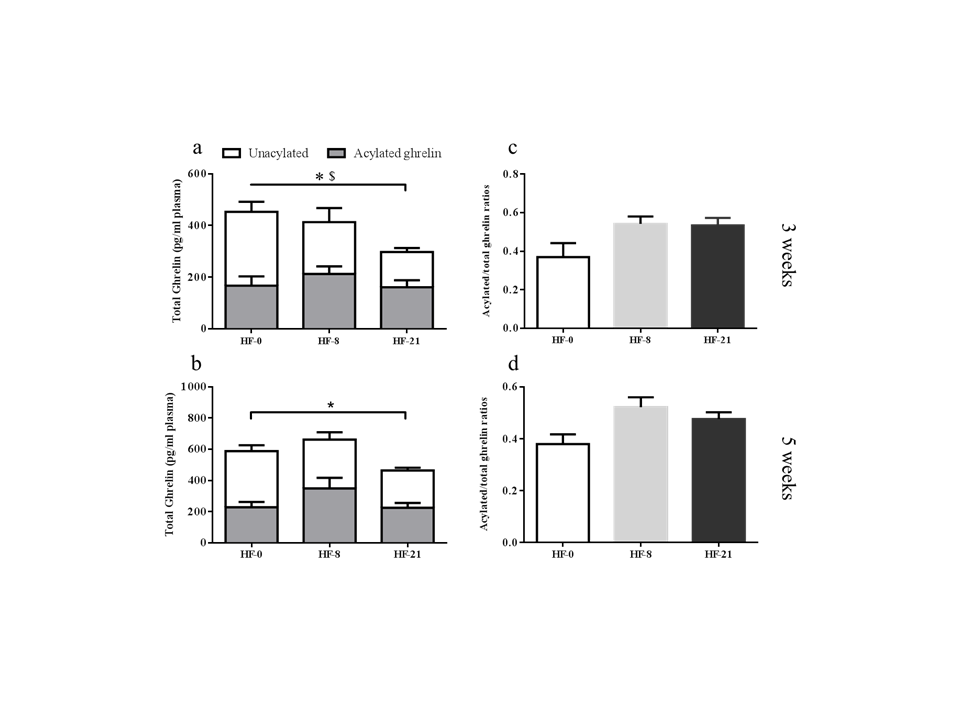

Supplement: S1 Fig — (a) Acylated and unacylated ghrelin concentrations after 3 weeks of diet. Kruskal-Wallis test (K-W): (i) acylated ghrelin, P = 0.201; (ii) unacylated ghrelin, P = 0.04, Dunn’s post tests, *P<0.05; (iii) total ghrelin, P = 0.033, Dunn’s post tests, $P<0.05. (b) Acylated and unacylated ghrelin concentrations after 5 weeks of diet. K-W test: (i) acylated ghrelin, P = 0.304; (ii) unacylated ghrelin, P = 0.023; Dunn’s post tests, *P<0.05; (iii) total ghrelin, P = 0.2938. (c) Acylated/total ghrelin ratios after 3 weeks of diet. K-W test: P = 0.0669. (d) Acylated/total ghrelin ratios after 5 weeks of diet. K-W test: P = 0.0653. (TIF) [file pone.0133600.s001.TIF]

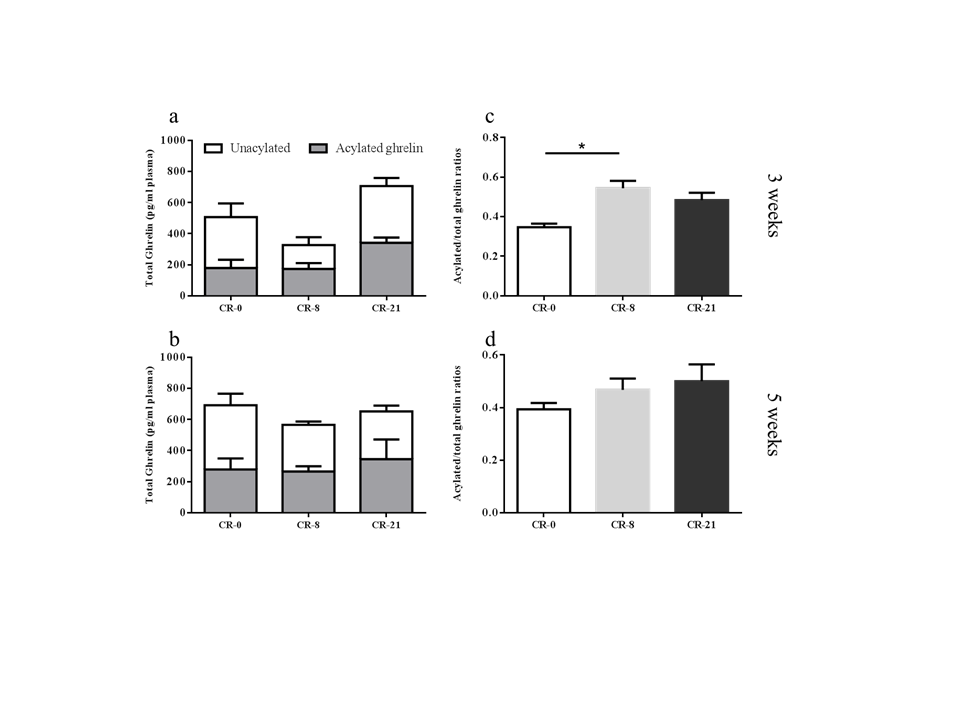

Supplement: S2 Fig — (a) Acylated and unacylated ghrelin concentrations after 3 weeks; Kruskal-Wallis test (K-W): (i) acylated ghrelin, P = 0.0714; (ii) unacylated ghrelin, P = 0.1321; (iii) total ghrelin, P = 0.1321. (b) Acylated and unacylated ghrelin concentrations after 5 weeks; K-W test: (i) acylated ghrelin, P = 0.9929; (ii) unacylated ghrelin, P = 0.6286; (iii) total ghrelin, P = 0.9929. (c) Acylated/total ghrelin ratios after 3 weeks; K-W test, P = 0.0250, Dunn’s post tests, *P<0.05. (d) Acylated/total ghrelin ratios after 5 weeks; K-W test, P = 0.2071. (TIF) [file pone.0133600.s002.TIF]

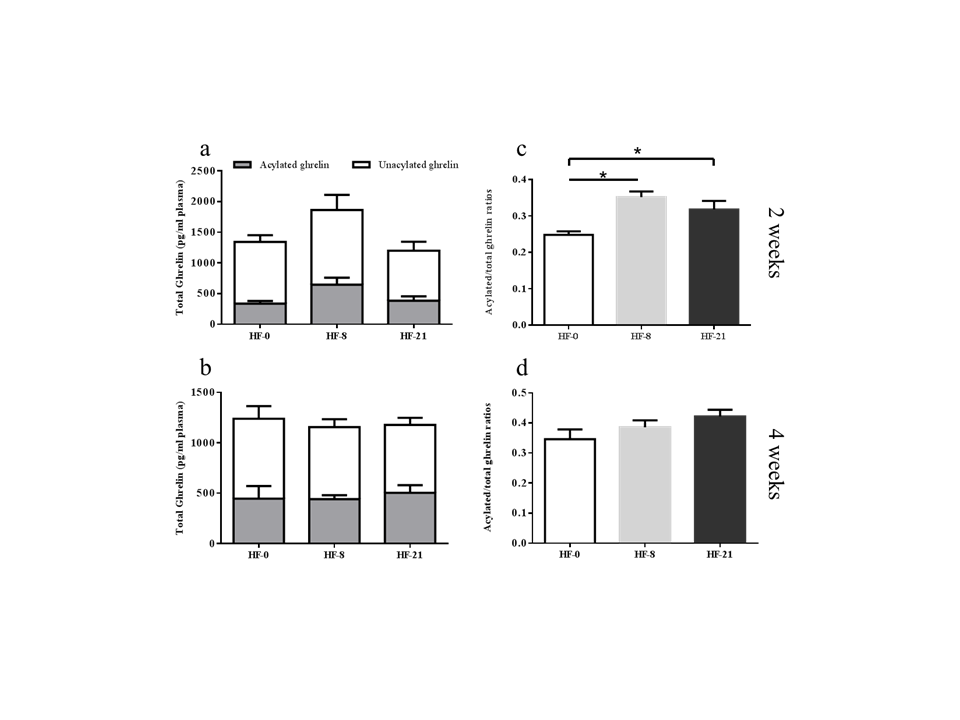

Supplement: S3 Fig — (a) Acylated and unacylated ghrelin concentrations after 2 weeks; Kruskal-Wallis test (K-W): (i) acylated ghrelin, P = 0.0958; (ii) unacylated ghrelin, P = 0.5380; (iii) total ghrelin, P = 0.3532. (b) Acylated and unacylated ghrelin concentrations after 4 weeks; K-W test: (i) acylated ghrelin, P = 0.6808; (ii) unacylated ghrelin, P = 0.8580; (iii) total ghrelin, P = 0.9826. (c) Acylated/total ghrelin ratios after 2 weeks; K-W test, P = 0.0076, Dunn’s post tests, *P<0.05, **P<0.01. (d) Acylated/total ghrelin ratios after 4 weeks, K-W test, P = 0.2248. (TIF) [file pone.0133600.s003.TIF]

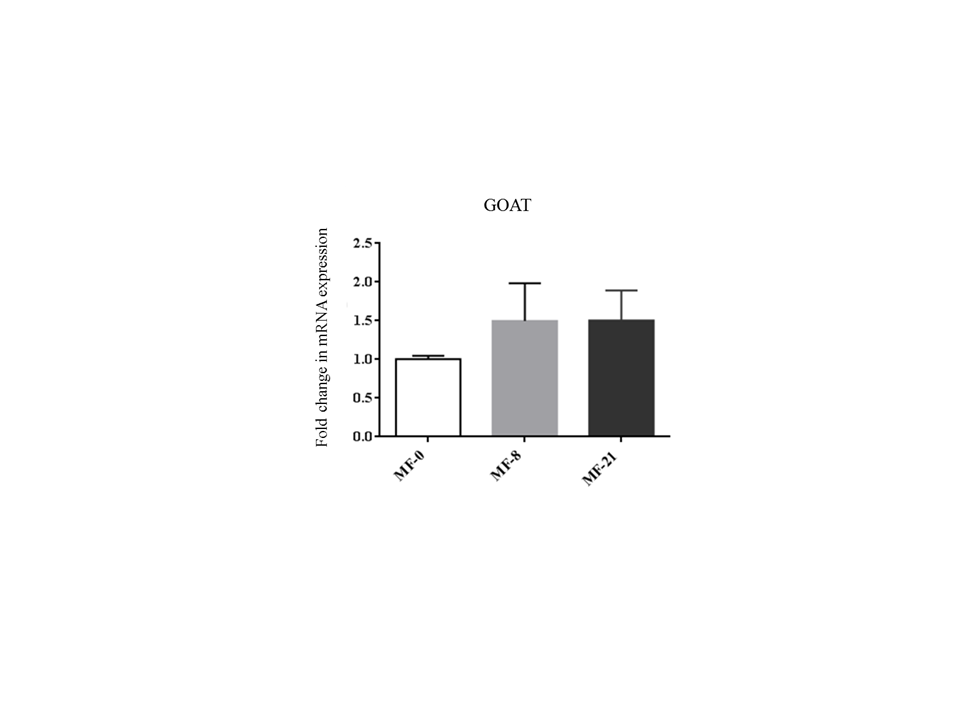

Supplement: S4 Fig — GOAT mRNA level (in fold change). K-W test, P>0.05. Results are expressed as mean ± SEM. (TIF) [file pone.0133600.s004.TIF]

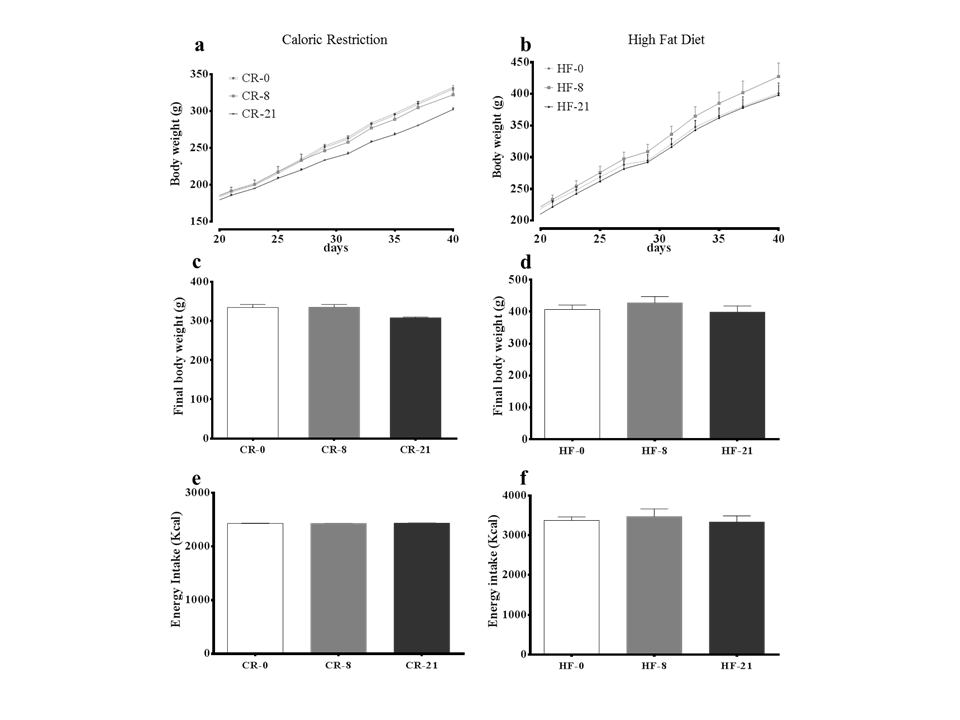

Supplement: S5 Fig — (a) Body weight gain (in gram) in CR groups. Two-way repeated measurement ANOVA: effect of the diet, P = 0.0662. (b) Body weight gain (in gram) in HF diets. Two-way repeated measurement ANOVA: effect of the diet, P = 0.5743. (c) Final Body weight (in gram) after 6 weeks on CR diets. Kruskal-Wallis test (K-W): P = 0.0714. (d) Final Body weight (g) after 6 weeks on HF diets. K-W test: P = 0.5097 (e) Cumulative energy intake after 6 weeks of CR diets (in Kcal); SEM is equal to zero because the rats ate their whole daily food ration; (f) Cumulative energy intake after 6 weeks on HF diets (in Kcal). K-W test: P = 0.9286 Results expressed as mean ± SEM. (TIF) [file pone.0133600.s005.TIF]

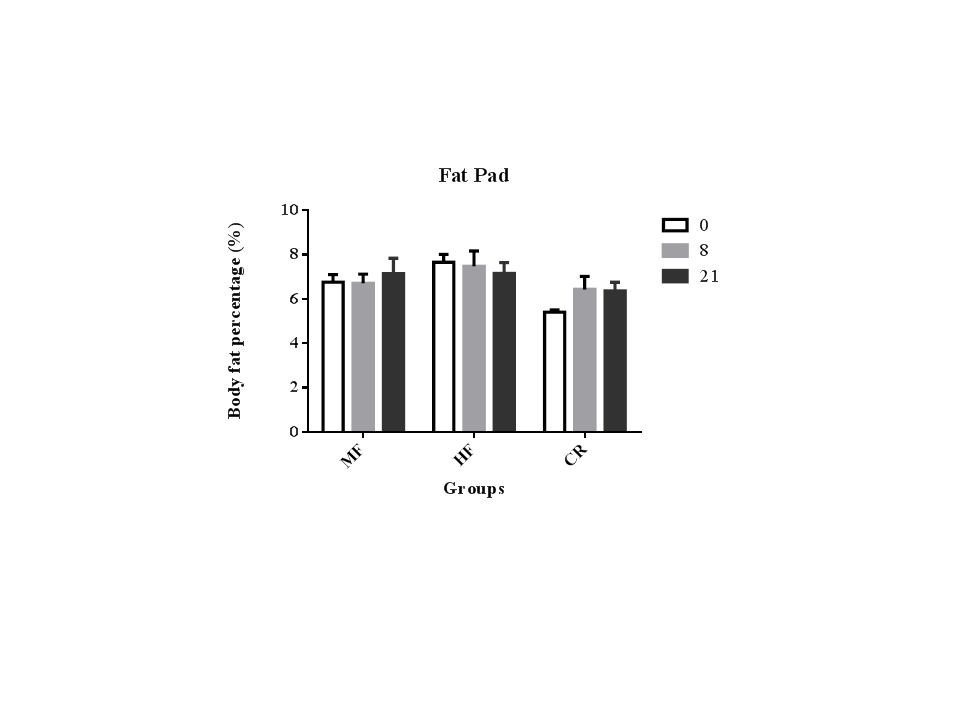

Supplement: S6 Fig — Masses of mesenteric, epididymal, subcutaneous and retroperitoneal relate to total body weight. K-W test: MF groups, P = 0.9384; HF groups, P = 0.6737; CR groups, P = 0.096. (TIF) [file pone.0133600.s006.TIF]

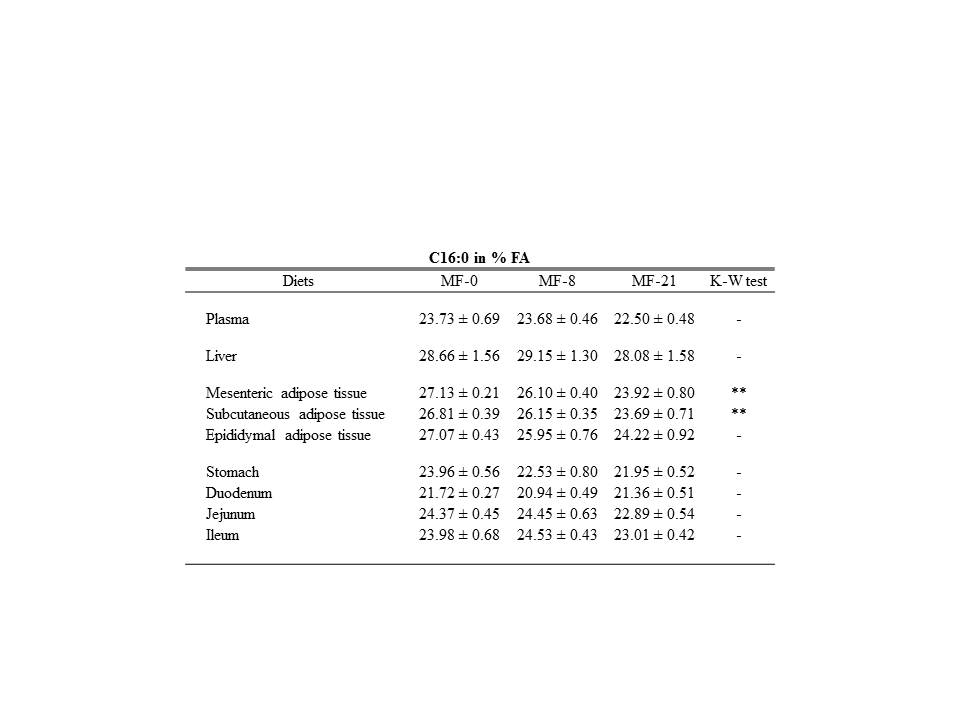

Supplement: S1 Table — Results expressed as mean ± SEM. (TIF) [file pone.0133600.s007.TIF]

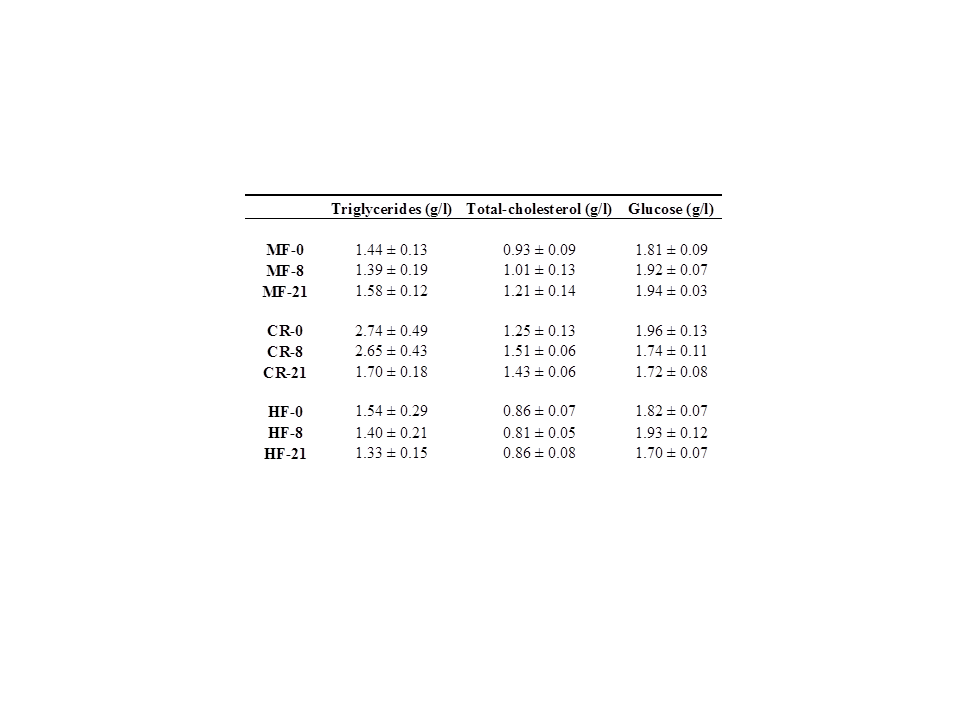

Supplement: S2 Table — Results expressed as mean ± SEM. (TIF) [file pone.0133600.s008.TIF]
